# A Retrospective Audit of Clinically Significant Maternal Bacteraemia in a Specialist Maternity Hospital from 2001 to 2014

**DOI:** 10.1155/2015/518562

**Published:** 2015-10-01

**Authors:** Richard John Drew, Zara Fonseca-Kelly, Maeve Eogan

**Affiliations:** ^1^Department of Clinical Microbiology, Rotunda Hospital, Dublin, Ireland; ^2^Department of Clinical Microbiology, Royal College of Surgeons in Ireland, Dublin, Ireland; ^3^Department of Obstetrics and Gynaecology, Rotunda Hospital, Dublin, Ireland

## Abstract

Maternal sepsis is a significant problem in obstetrics, with almost one in four maternal deaths related to severe sepsis. We carried out a retrospective review of clinically significant bacteraemia in obstetric patients attending Rotunda Hospital over 14 years. From 2001 to 2014, there were 252 clinically significant positive blood culture episodes in obstetric patients. There were 112,361 live births >500 g during the study period giving an overall rate of 2.24 clinically significant positive maternal blood culture episodes per 1000 live births >500 g. The median rate over the 14 years was 2.12 episodes per 1000 live births >500 g, with an interquartile range of 1.74–2.43 per 1000 live births >500 g. There was no discernable increasing or decreasing trend over the 14 years. *E. coli* was the most commonly isolated organism (*n* = 92/252, 37%), followed by group B *Streptococcus* (*n* = 64/252, 25%), *Staphylococcus aureus* (*n* = 28/252, 11%), and anaerobes (*n* = 11/252, 4%). These top four organisms represented three-quarters of all positive blood culture episodes (*n* = 195/252, 77.3%). Of note, there were only five cases of listeriosis, representing a rate of 4.4 cases per 100,000 live births >500 g. The rate of invasive group A streptococcal infection was also very low at 5.3 cases per 100,000 live births >500 g.

## 1. Introduction

Mortality and morbidity due to maternal sepsis still remain a significant issue in modern obstetrics. As shown in the most recent confidential enquiry into maternal deaths in the UK and Ireland, one in four mothers that died, died as a result of severe sepsis [1]. It is thus crucial to understand the epidemiology and incidence of maternal bacteraemia, in order to improve the care provided to mothers with sepsis. A large study of almost 45 million deliveries in the United States of America showed that sepsis complicated 1 in every 3333 deliveries, with severe sepsis being present in 1 in every 10,823 deliveries [2]. Of concern in this paper was that there was a recent statistically significant increase in severe sepsis and sepsis related death, primarily occurring in mothers who had complex preexisting medical conditions such as chronic renal or cardiac disease.

With this increase in maternal mortality and severe sepsis, it is necessary to monitor trends in maternal sepsis and use the information to identify key targets for improvement in the future. The purpose of this study was to determine the rate of clinically significant maternal bacteraemia in patients attending Rotunda Hospital in Dublin, which is a large maternity hospital with approximately 9,000 births a year. A secondary aim was to identify the organisms that are responsible for the bacteraemia episodes and to determine if there has been a change over time.

## 2. Materials and Methods

This was a retrospective audit of all clinically significant positive blood cultures in obstetric patients taken at the Rotunda Hospital, Dublin, from 2001 to 2014. Rotunda Hospital is a stand-alone maternity hospital which serves a large catchment area in the north part of Dublin and surrounding regions. It provides specialist obstetric care and has a large perinatal medicine centre. The primary purpose of this study was to determine the rate of clinically significant positive blood cultures in obstetric patients, expressed per 1000 live births >500 g. The secondary purpose was to determine which organisms caused the clinically significant bacteraemia events and if their prevalence had changed over time. This study was approved by the Ethics Committee of Rotunda Hospital (approval number RAG-2015-005).

Data was collected retrospectively from the laboratory information system. During the study period, the BacTAlert (BioMerieux, France) system was used and the machine was upgraded as new versions were brought to market. Initially, all positive blood cultures were identified from the laboratory information system. Organisms which are classically associated as being pathogens (e.g.,* E*.* coli* and* S*.* aureus*) were included in the analysis. Organisms that are classically considered contaminants (e.g., coagulase negative staphylococci, diphtheroids) as per the CDC definitions were only included if there were two or more positive blood cultures occurring within 48 hours [3]. All positive blood cultures in the same patient that occurred within 14 days were considered to be the same episode, again in line with CDC definitions. Information was organised and analysed using Microsoft Excel. Each patient episode was reviewed to ensure that the patient had presented as an obstetric patient rather than a gynaecology patient.

There was potential bias in that this was a retrospective study; however, by using the electronic laboratory system which was in place during the period of the study it, was possible to ensure that all positive blood cultures were included.

## 3. Results and Discussion

From 2001 to 2014, there were 830 positive blood cultures taken from 799 patients. Repeat positive blood cultures were considered to represent one episode if they occurred within 14 days, and also contaminants were removed in line with the CDC definitions described in the methods. This left 262 clinically significant positive blood culture episodes. Ten were excluded as further review identified them as gynaecology patients, leaving a total of 252 clinically significant positive blood culture episodes in obstetric patients over the period studied.

**Figure 1:**
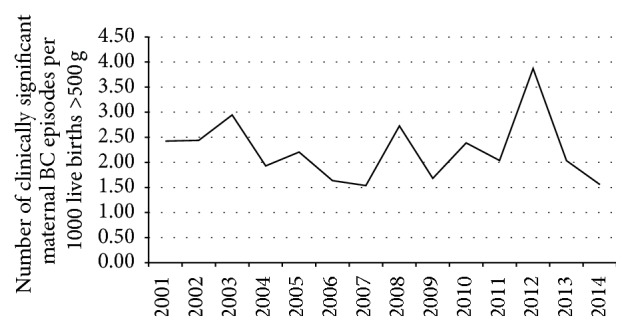
Rate of clinically significant obstetric related positive blood culture episodes per 1000 live births >500 g in Rotunda Hospital from 2001 to 2014.

There were 112,361 live births >500 g during that period giving an overall rate of 2.24 clinically significant positive maternal blood culture episodes per 1000 live births >500 g. The median rate was 2.12 episodes per 1000 live births >500 g, with an interquartile range of 1.74–2.43 per 1000 live births >500 g (range 1.54–3.87 per 100 live births >500 g). The rate was static and there was no discernable trend over the 14 years (Figure 1).

*E*.* coli* was the most commonly isolated organism (*n* = 92/252, 37%), followed by group B* Streptococcus* (*n* = 64/252, 25%),* Staphylococcus aureus* (*n* = 28/252, 11%), and anaerobes (*n* = 11/252, 4%). These top four organisms represented three-quarters of all positive blood culture episodes (*n* = 195/252, 77.3%). Twenty-five of the* S*.* aureus* positive blood cultures were methicillin-sensitive (*n* = 25/28, 89.2%) and three were methicillin-resistant (*n* = 3/28, 10.8%). The full results are detailed in Table 1. Of note, there were only five cases of listeriosis, representing a rate of 4.4 cases per 100,000 live births >500 g. The rate of invasive group A streptococcal infection was also very low at 5.3 cases per 100,000 live births >500 g. There was only one invasive case of Candidiasis isolated during the study period.

## 4. Conclusions

The clinically significant bacteraemia rate of 2.12 episodes per 1000 live births >500 g is comparable to the rate found in other studies carried out in Dublin, which found that the rate of bacteraemia was 1.81 per 1000 pregnancies and also 1.5 per 1000 live births >500 g [4, 5]. A French study reviewing data from 2005 to 2009 showed that the bacteraemia rate in maternity cases was 2.8 per 1000 deliveries, which was higher than in our cohort [6]. This rate is also roughly equivalent to that found in a Scottish based population case control study of maternal bacteraemia, in which the most recent three-year rate of maternal bacteraemia was found to be 1.65 per 1000 pregnancies [7]. The predominance of* E*.* coli* and group B* Streptococcus* as pathogens has been demonstrated in other studies and is a recurrent theme [4, 6].

Although classically described as causes of peripartum bacteraemia in pregnant women, both listeriosis (4.4 per 100,000 deliveries with a live birth >500 g) and group A* Streptococcus* (5.3 per 100,000 live births >500 g) were actually very rare in our patient population. The low incidence of listeriosis may be due to improved maternal education regarding at-risk dairy products and the low rate of consumption of unpasteurised dairy produce in Ireland. There is very little international data on the rates of perinatal listeriosis as it is a rare disease. A study of fetomaternal listeriosis in Denmark from 1981 to 88 showed that the rate varied from 1 per 100,000 live births up to 25.3 per 100,000 live births depending on whether or not there was an epidemic occurring at the time of an indistinguishable strain of listeria [8]. Within our cohort, the rate of group A* Streptococcus* is similar to the rate in a population wide study of maternal group A* Streptococcus* bacteraemia in the United States of America from 1995 to 2000, which found the rate to be 6 per 100,000 live births [9].

In terms of bias, the main issue with this study is that it is a retrospective audit, using laboratory definitions to determine if the blood culture was clinically significant or not. This may give an artificially high rate but does provide a useful indicator of the overall rate of bacteraemia, which allows international comparisons to be made. Retrieval of organisms from positive blood cultures has improved during the time of the study as the machines which monitor positive blood cultures have become more sensitive; however, it would not be expected to impact greatly this study as the key pathogens (*E*.* coli, S*.* aureus*, and anaerobes) are easily recovered from positive blood cultures.

This data shows that despite many advances in the management of perinatal sepsis, including intrapartum antimicrobial prophylaxis and improved diagnostics, the key pathogens still remain* E*.* coli*, group B* Streptococcus*, and* S*.* aureus*. Thus, it is important to still focus on these organisms and specifically to optimise care for women with chorioamnionitis and urinary tract infections, which are the likely aetiologies of bacteraemia in pregnant women. Although the data presented here cannot be generalised to all settings, it has been shown to be equivalent to studies carried out in other developed maternity settings.

There is a need for maternity services to prioritise sepsis surveillance programmes on a national basis; these can then be integrated with surveillance programmes across Europe, primarily to optimise care of pregnant women but also to monitor trends as they emerge. As the incidence of maternal bacteraemia is so low, particularly with regard to certain organisms such as listeriosis and group A* Streptococcus*, this would enable national and international collaboration so that trends may be identified earlier, rather than relying on historical cohorts. This would enable development of responsive interventions and allow a real-time analysis of the impact of maternal sepsis strategies, to ensure that they lead to a better outcome for patients.

## Figures and Tables

**Table 1:** Overview of clinically significant obstetric related positive blood cultures in Rotunda Hospital from 2001 to 2014.

Rotunda significant positive blood cultures	2001	2002	2003	2004	2005	2006	2007	2008	2009	2010	2011	2012	2013	2014	Total	%
*Escherichia coli*	5	5	5	3	8	5	2	7	7	9	7	13	8	8	92	37%
Group B *Streptococcus*	5	5	1	3	4	2	1	3	5	6	6	14	5	4	64	25%
*S. aureus* (methicillin-sensitive)	2	2	4	3	1	0	3	3	1	2	2	0	2	0	25	10%
*S. aureus* (methicillin-resistant)	0	1	0	0	1	0	0	0	0	0	1	0	0	0	3	1%
Anaerobes	1	0	0	1	0	0	2	1	0	2	2	2	0	0	11	4%
*Enterococcus* spp.	0	0	1	1	0	1	1	2	0	0	0	1	1	0	8	3%
*Listeria*	0	1	1	0	0	0	2	1	0	0	0	0	0	0	5	2%
Group A *Streptococcus*	0	0	0	0	0	0	0	1	0	0	0	3	1	1	6	2%
*Acinetobacter* spp.	0	0	2	0	1	0	1	0	0	0	0	0	0	0	4	2%
*Haemophilus influenzae*	0	0	0	0	0	0	1	0	0	0	1	1	1	0	4	2%
Mixed	1	0	0	0	0	1	0	1	0	1	0	0	0	0	4	2%
*Streptococcus pneumoniae*	0	0	0	0	0	2	0	1	0	0	0	0	0	0	3	1%
*Klebsiella* spp.	0	0	1	0	0	0	0	0	1	0	0	0	0	0	2	1%
Group F *Streptococcus*	0	1	0	0	0	0	0	2	0	0	0	0	0	0	3	1%
*Proteus* spp.	1	1	1	0	0	0	0	0	0	0	0	0	0	0	3	1%
*Citrobacter* spp.	1	0	1	1	0	0	0	0	0	0	0	0	0	0	3	1%
*Burkholderia cepacia*	0	0	0	0	0	0	0	1	0	0	0	0	0	1	2	1%
*Salmonella*	0	0	2	0	0	0	0	0	0	0	0	0	0	0	2	1%
*Clostridium* spp.	0	0	0	0	0	1	0	0	1	0	0	0	0	0	2	1%
*Streptococcus anginosus*	0	0	1	0	0	0	0	0	0	0	0	0	0	0	1	0.4%
*Morganella morganii*	0	0	0	1	0	0	0	0	0	0	0	0	0	0	1	0.4%
*Pseudomonas aeruginosa*	0	0	0	0	0	0	0	1	0	0	0	0	0	0	1	0.4%
Group C *Streptococcus*	0	1	0	0	0	0	0	0	0	0	0	0	0	0	1	0.4%
*Candida glabrata*	0	0	0	0	0	0	0	0	0	0	0	1	0	0	1	0.4%
*Streptococcus* spp.	0	0	0	0	0	0	0	0	0	1	0	0	0	0	1	0.4%
Total	16	17	20	13	15	12	13	24	15	21	19	35	18	14	252	100%
